# Comparative analyses of chloroplast genomes from Six *Rhodiola* species: variable DNA markers identification and phylogenetic relationships within the genus

**DOI:** 10.1186/s12864-022-08834-9

**Published:** 2022-08-11

**Authors:** Kaihui Zhao, Lianqiang Li, Hong Quan, Junbo Yang, Zhirong Zhang, Zhihua Liao, Xiaozhong Lan

**Affiliations:** 1 The Provincial and Ministerial Co-Founded Collaborative Innovation Center for R & D in Tibet Characteristic Agricultural and Animal Husbandry Resources, The Center for Xizang Chinese (Tibetan) Medicine Resource, Joint Laboratory for Tibetan Materia Medica Resources Scientific Protection and Utilization Research of Tibetan Medical Research Center of Tibet, Tibet Agriculture and Animal Husbandry University, Nyingchi, 860000 Tibet China; 2 Key Laboratory of Forest Ecology in Tibet Plateau, Ministry of Education, Tibet Agricultural & Animal Husbandry University, Nyingchi, 860000 Tibet China; 3 Integrative Science Center of Germplasm Creation, The Provincial and Ministerial Co-Founded Collaborative Innovation Center for R & D in Tibet Characteristic Agricultural and Animal Husbandry Resources, SWU-TAAHC Medicinal Plant Joint R&D Centre, School of Life Sciences, Southwest University, Chongqing, 400715 China; 4 Germplasm Bank of Wild Species, Kunming Institute of Botany, Chinese Academy of Sciences, Kunming, 650201 Yunnan China

**Keywords:** *Rhodiola*, Chloroplast genome, Divergent hotspots, Phylogenetic analysis

## Abstract

**Background:**

As a valuable medicinal plant, *Rhodiola* has a very long history of folk medicine used as an important adaptogen, tonic, and hemostatic. However, our knowledge of the chloroplast genome level of *Rhodiola* is limited. This drawback has limited studies on the identification, evolution, genetic diversity and other relevant studies on *Rhodiola*.

**Results:**

Six *Rhodiola* complete chloroplast genomes were determined and compared to another *Rhodiola* cp genome at the genome scale. The results revealed a cp genome with a typical quadripartite and circular structure that ranged in size from 150,771 to 151,891 base pairs. High similarity of genome organization, gene number, gene order, and GC content were found among the chloroplast genomes of *Rhodiola*. 186 (*R. wallichiana*) to 200 (*R. gelida*) SSRs and 144 pairs of repeats were detected in the 6 *Rhodiola* cp genomes. Thirteen mutational hotspots for genome divergence were determined and could be used as candidate markers for phylogenetic analyses and *Rhodiola* species identification. The phylogenetic relationships inferred by members of *Rhodiola* cluster into two clades: dioecious and hermaphrodite. Our findings are helpful for understanding *Rhodiola*'s taxonomic, phylogenetic, and evolutionary relationships.

**Conclusions:**

Comparative analysis of chloroplast genomes of *Rhodiola* facilitates medicinal resource conservation, phylogenetic reconstruction and biogeographical research of *Rhodiola*.

**Supplementary Information:**

The online version contains supplementary material available at 10.1186/s12864-022-08834-9.

## Introduction

As traditional natural plant pharmaceuticals and health food, *Rhodiola* belongs to the family Crassulaceae and mainly distributed in alpine regions of Asia and Europe [1–3]. 73 species of *Rhodiola* plants are distributed in China, and the Qinghai-Tibet Plateau has the most species [4]. The extract of *Rhodiola* plants, especially *R. crenulata* and *R. rosea*, has various pharmacological effects such as anti-hypoxia, fatigue, tumors, radiation, aging, and improvement of mental and physical functions [5]. Due to the fragile ecological environment of the Qinghai-Tibet Plateau and the lack of artificial cultivation techniques, relying solely on the digging of wild resources can easily lead to the reduction of *Rhodiola* plant resources and the loss of genetic diversity resources [6].

Due to the variety of *Rhodiola* plants, the source of commercial medicine of *Rhodiola* is very complicated, but the pharmacodynamics of different species of *Rhodiola* have a significant difference in clinical efficacy [7]. The traits of the medicinal plants of *Rhodiola* and the characteristics of the microstructure are similar [7]. At present, *R. crenulata* is the only primordial plant of the Rhodiola medicinal herbs contained in the Chinese Pharmacopoeia (2020 Edition). The development and research of its alternative varieties is imminent. In recent years, the mixed use of plant roots and rhizomes of distinct species of *Rhodiola* has occurred frequently. Researchers have studied the Rhodiola Herbal Slices on the market using DNA barcoding technology, only 40% of the samples are *R. crenulata* collected in the Chinese Pharmacopoeia [8]. The mixuse of *Rhodiola* medicinal materials directly affects the safety and efficacy of clinical medications, and coupled with unrestricted collection, the number of wild resources has decreased dramatically. Therefore, in order to realize the protection and sustainable development of *Rhodiola* plants, it is necessary to conduct in-depth research on the identification and genetic diversity of their species.

To solve the problem of *Rhodiola* plant identification, Wang et al. developed random amplification polymorphic DNA (RAPD) and inter-simple sequence repeat (ISSR) primers to identify *R. angusta, R. crenulata, R. bupleuroides,* and *R. sachalinensis* [9]. Li et al. established a method for classifying and identifying *R. quadrifida* and *R. crenulata* based on nuclear magnetic resonance 1H-NMR fingerprints-chemical pattern recognition technique [10]. Zhu et al.found that the internal transcribed spacer 2 (ITS2) sequence can effectively distinguish *R. crenulata* and *R. rosea* [11]. Booker et al. used nuclear magnetic resonance spectroscopy coupled with high performance thin layer chromatography techniques to comprehensively analyze *R. crenulata* and *R. rosea* collected in markets around the world [12]. These advanced identification methods currently used can solve the identification problems of some *Rhodiola* plants, but they also have the disadvantages of having a narrow application range and high identification cost.

DNA barcoding is a new species identification technology developed in recent years [13]. It eliminates the obstacles of traditional morphological recognition methods that rely on long-term experience. The plant chloroplast (cp) genome, as a research hotspot for screening DNA barcoding sequences, can also be used as a super-barcoding for phylogenetic and species identification studies [14]. The use of the cp genome to solve the problem of difficult classification of related species is of great significance for species identification in herbal medicine and even the entire plant community. Chen et al. proposed that using the whole genome as a super-barcode can effectively identify *Ligularia* plants [15]. Zhong et al. found that 41 *Dendrobium* species can be effectively identified based on the whole cp genome, and *Dendrobium officinale* from 3 different places of production can also be distinguished [16].

For a long time, due to the difficulties in collecting samples and specimens, the phylogenetic relationship within *Rhodiola* is still poorly understood [17]. Mayuzumi et al. proposed that *Rhodiola* as a distinct genus from *Sedum* and a close relationship between *Rhodiola* and *Pseudosedum* [3]. A recent molecular phylogenetic study using plastome genomes sampled about 12 representative species of *Rhodiola* supported *Rhodiola* was divided into dioecious clade and hermaphrodite clade [4]. Although these studies provided new and important insights into the phylogeny of *Rhodiola*, a broader sampling scheme is needed to better understand the phylogenetic relationships of *Rhodiola*.

In this study, we sequenced the cp genomes of *R. tangutica, R. wallichiana, R. quadrifida, R. bupleuroides, R. gelida,* and *R. henryi* using Illumina technology followed by reference-guided assembly of de novo contigs. Our aims were: 1) to detect the variations of long repeats and SSRs in 6 *Rhodiola* cp genomes; 2) to identify divergence hotspots as potential genetic markers for *Rhodiola* DNA barcoding; and 3) to reconstruct a phylogeny for *Rhodiola* species using protein coding sequences of the cp genome and infer their phylogenetic location within *Crassulaceae*.

## Results

### General features of the Six *Rhodiola* cp genomes

**Fig. 1:**
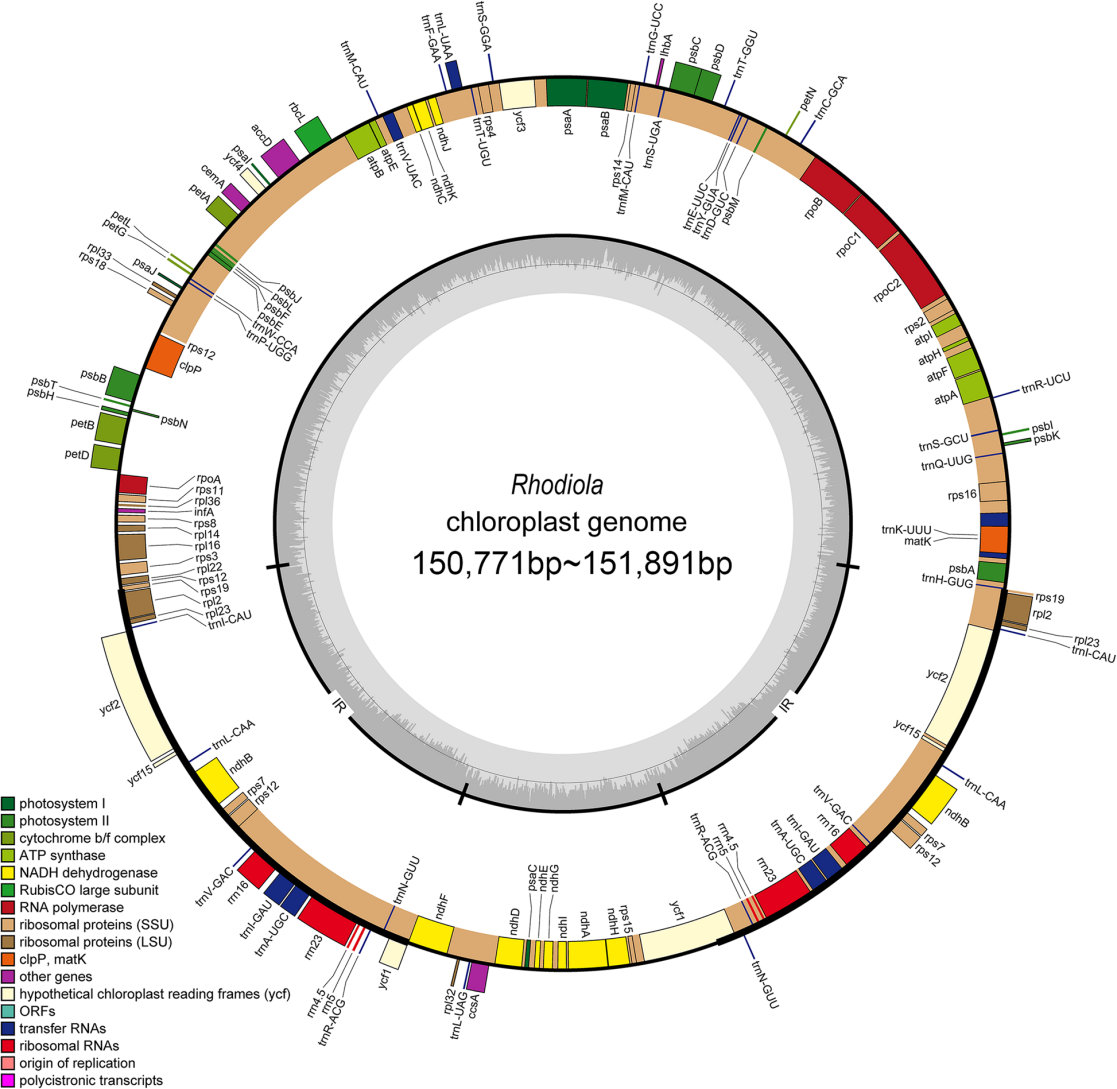
Gene maps of the complete cp genome of six species of *Rhodiola*. Genes drawn outside of the map circle are transcribed clockwise, while those drawn inside are transcribed counter-clockwise. The darker gray in the inner circle corresponds to GC content while the lighter gray corresponds to AT content

The cp genomes of *R. tangutica* (2.4 Gb)*, R. wallichiana* (2.1 Gb)*, R. quadrifida* (2.4 Gb)*, R. bupleuroides* (2.2 Gb)*, R. gelida* (2.1 Gb)*,* and *R. henryi* (2.3 Gb) were sequenced with approximately 2.0 Gb of paired-end reads, respectively. Clean reads were achieved by removing adaptors and low-quality read pairs. The recovered clean reads for *R. quadrifida*, *R. tangutica, R. wallichiana*, *R. bupleuroides*, *R. gelida*, and *R. henryi* were 1,737,149, 1,013,832, 839,613, 973,418, 866,547, and 1,092,141, respectively (Table S1). Six *Rhodiola* complete cp genome maps (Fig. 1) were obtained through de novo genome sequencing and assembly with the reference *R. rosea* (MH410216) genome. The average organelle coverage for *R. quadrifida*, *R. tangutica, R. wallichiana*, *R. bupleuroides*, *R. gelida*, and *R. henryi* with the reference genome reached 1,378, 262, 254, 193, 203, and 313, respectively (Table S1).

The cp genome size ranged from 150,771 bp in *R. quadrifida* to 151,891 bp in *R. henryi*, which included 82,211 bp (*R. tangutica*) to 83,095 bp (*R. gelida*) large single-copy (LSC) regions, and 16,991 bp (*R. quadrifida*) to 17,104 bp (*R. tangutica*) small single-copy (SSC) regions, separated by a pair of 25,773 bp (*R. quadrifida*) to 25,887 bp (*R. henryi*) inverted repeat (IR) regions (Fig. 1; Table S1). There were 85 protein-coding genes, 37 tRNA genes, and 8 rRNA genes that were identified in each *Rhodiola* cp genome (Table S2). Among these unique genes, 15 genes harbored one intron and three genes (*ycf3*, *clpP*, and *rps12*) harbored two introns (Table S2).

### Long repeats and SSRs

**Fig. 2:**
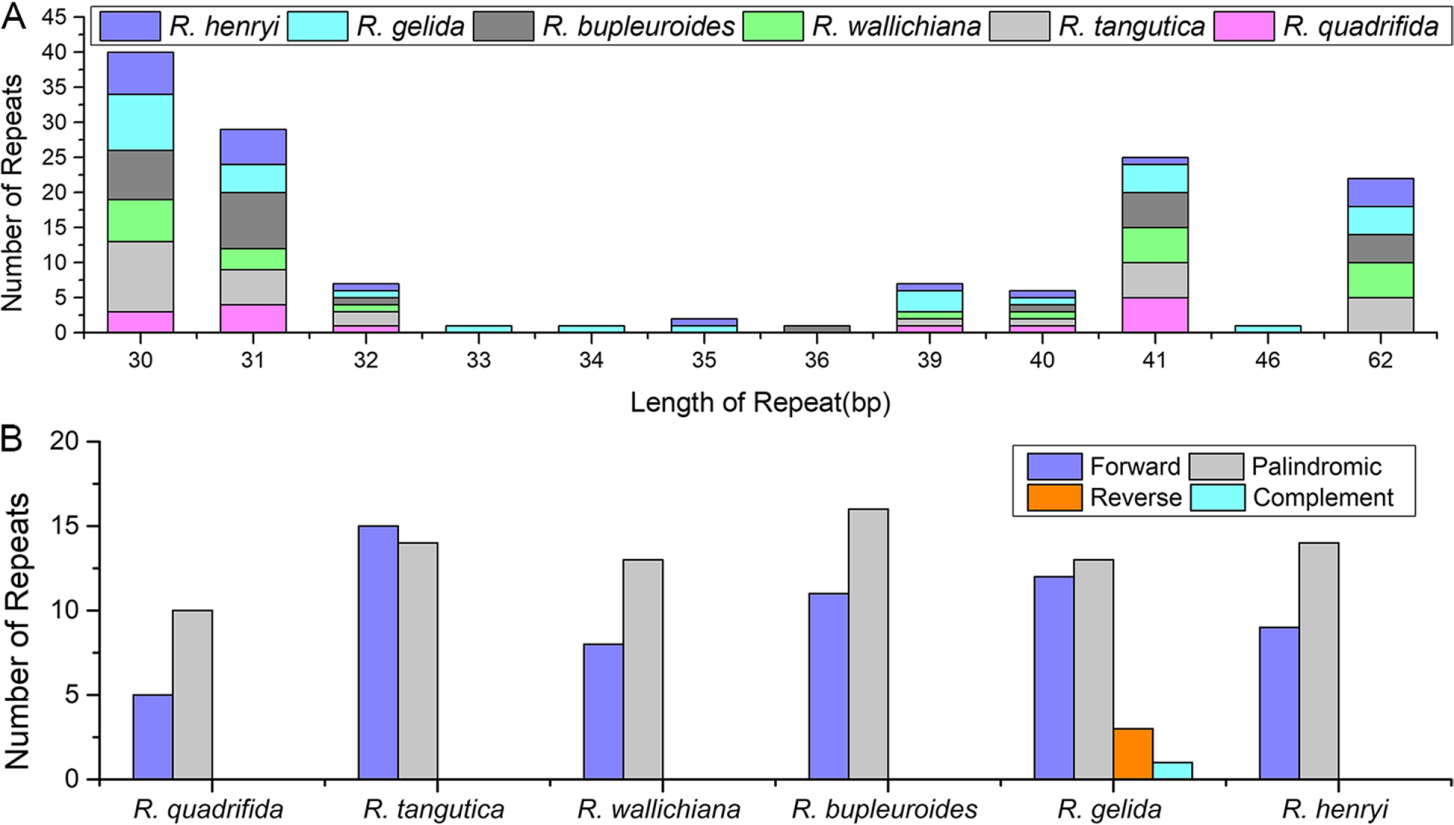
The number of long repeats in the whole cp genome sequence of the 6 *Rhodiola* species. **A** Frequency of repeats more than 30 bp long. **B** Frequency of repeat type

Repeat sequences have been applied extensively for phylogeny, population genetics, genetic mapping, and forensic studies [18]. A total of 144 pairs of repeats were detected in the 6 *Rhodiola* cp genomes, with the repeat length range from 30 to 62 bp (Fig. 2A). The cp genomes of 6 *Rhodiola* have 5, 15, 8, 11, 12 and 9 forward repeats and 10, 14, 13, 16, 13 and 14 palindromic repeats (Fig. 2B). Reverse repeats and complementary repeats only exist in the cp genome of *R. gelida* (Fig. 2B)*.* The long repeat lengths of 30, 31, 32, 40, and 41 bp existed in all 6 *Rhodiola* cp genomes (Fig. 2A). Long repeat lengths of 33, 34, and 36 bp were found the least often and only existed in the *R. gelida* and *R. bupleuroides* cp genomes, respectively (Fig. 2A).

**Fig. 3:**
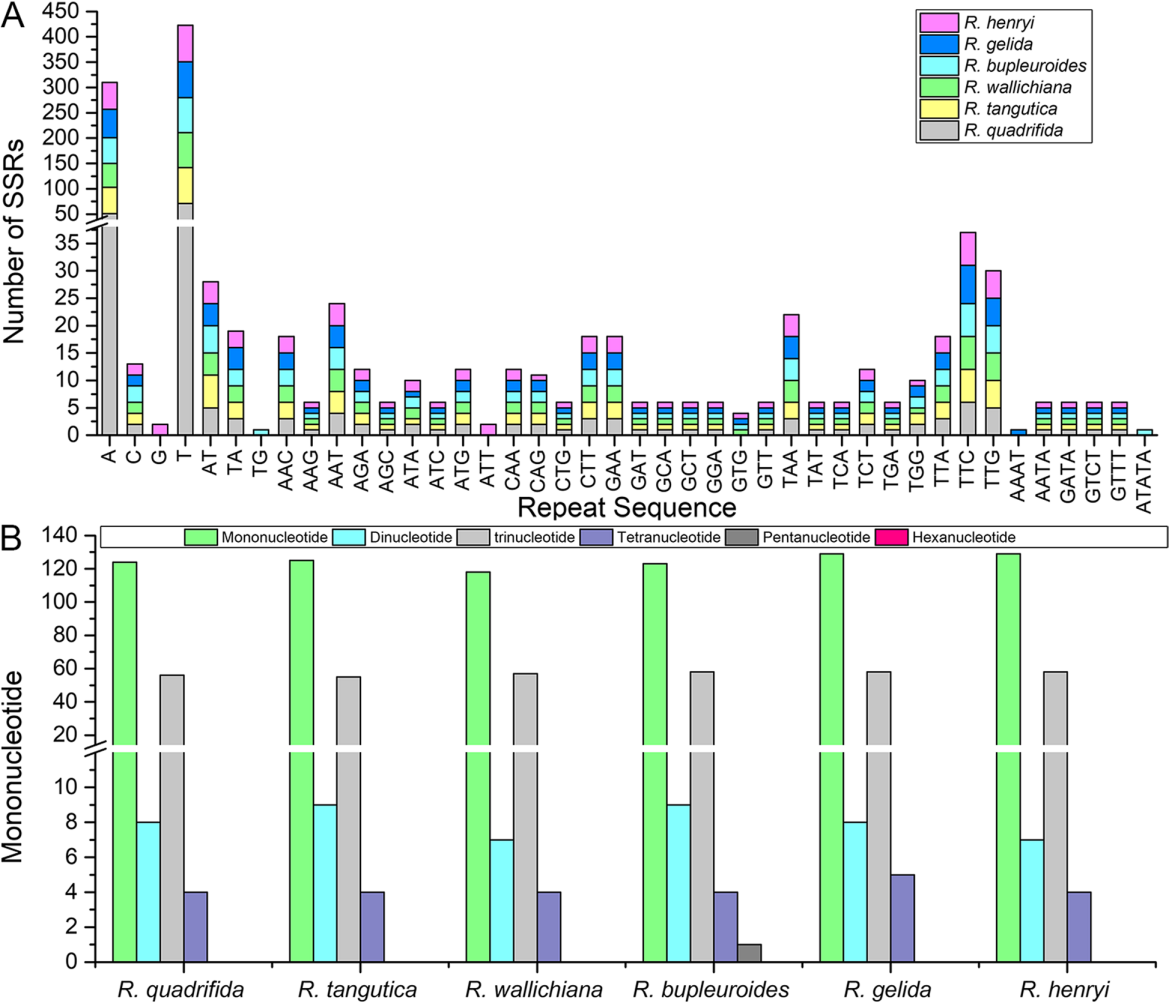
Analysis of SSRs in the 6 *Rhodiola* cp genomes. **A** Frequency of common motifs in the 6 *Rhodiola* cp genomes. **B** Number of different SSR types detected in the 6 *Rhodiola* cp genomes

Simple sequence repeats (SSRs) are usually 1–6 bp tandem repeat DNA sequences and are widely used as molecular markers with their polymorphic to identify closely related species [19]. In our study, SSRs in 6 *Rhodiola* cp genomes were identified using MISA software. The number of SSRs in the 6 *Rhodiola* species ranged from 186 (*R. wallichiana*) to 200 (*R. gelida*) (Fig. 3A). The numbers and distribution of all SSR types were similar and conserved in the 6 *Rhodiola* cp genomes, except for pentanucleotide, which only existed in *R. bupleuroides* (Fig. 3B). Mononucleotide repeat motifs were occupied the largest proportion in these SSRs, ranging from 63% (*R. bupleuroides*) to 65% (*R. henryi*). No hexanucleotide repeat motifs were found in the 6 *Rhodiola* cp genomes (Fig. 3B).

### Divergence hotspots

**Fig. 4:**
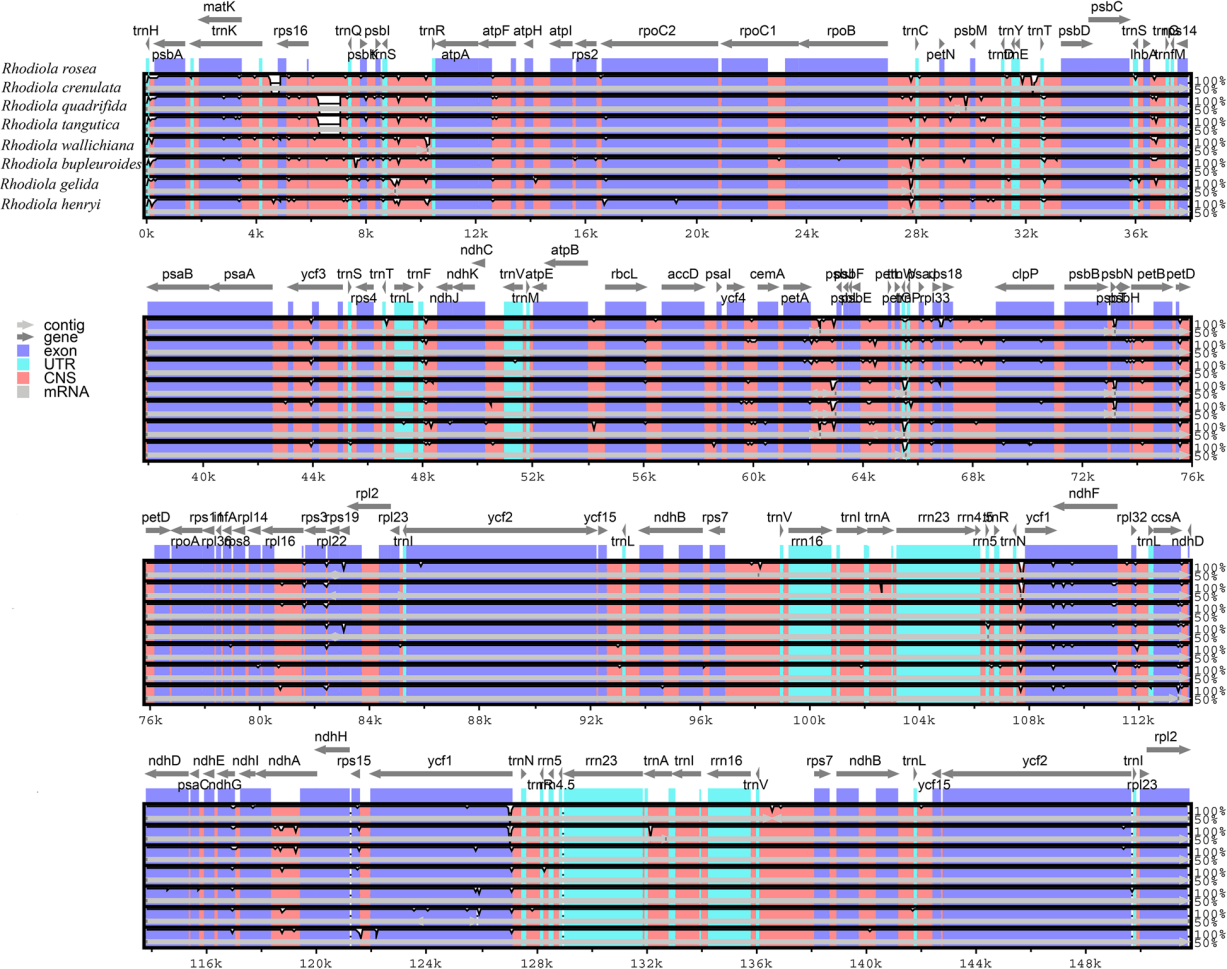
Sequence alignment of chloroplast genomes of eight *Rhodiola* species. Sequence identity plot comparing the chloroplast genomes with *R. rosea* as a reference using mVISTA. The grey arrows and thick black lines above the alignment indicate genes with their orientation. The Y-axis represents the identity from 50 to 100%

We selected eight *Rhodiola* cp genome sequences to be compared and plotted using the mVISTA software with the annotated cp genome of *R. rosea* as a reference to elucidate the level of sequence divergence (Fig. 4). Based on the overall sequence identity, the results indicated that the coding regions exhibit lower divergence levels than the non-coding regions and two IR regions exhibit higher conservation than the remaining sequences across the whole chloroplast genome, as can be seen in other plants [20, 21]. Furthermore, the results showed that the *ycf1* and *trnH-GUG-psbA* sequences of *Rhodiola* were highly divergent regions.

**Fig. 5:**
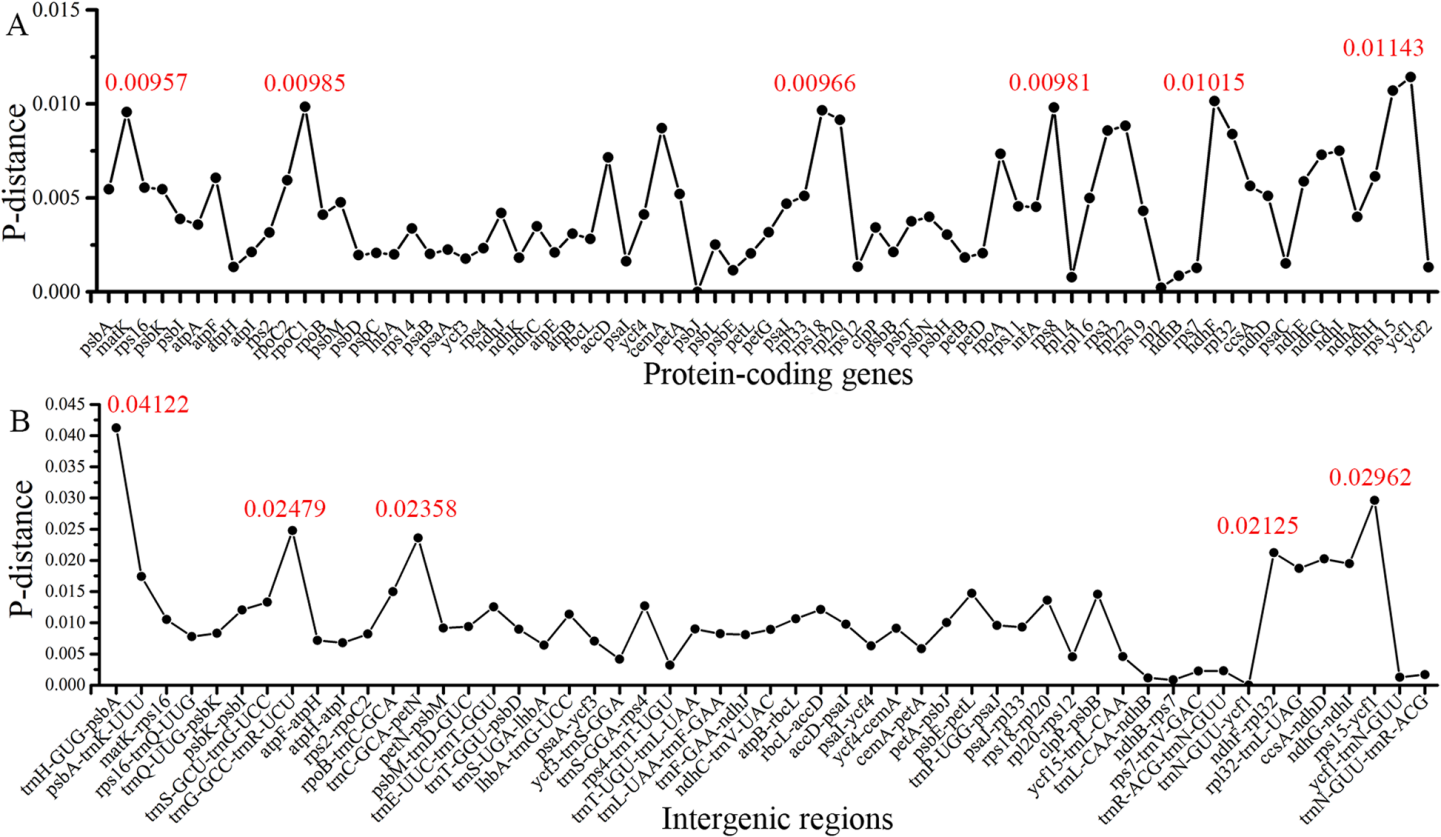
The nucleotide variability (*Pi*) values were compared among 22 *Rhodiola* species. **A** The P-distance value of protein-coding genes. **B** The P-distance value of intergenic regions

In addition, the nucleotide diversity (Pi) values were calculated to evaluate the sequence divergence among 22 *Rhodiola* cp genomes (Table S3). The genetic distance of all 75 protein-coding genes varied from 0 to 0.01143 (*ycf1*) with an average value of 0.00444 (Fig. 5A). Based on a considerably higher Pi value of > 0.009, we found seven highly variable regions (*ycf1*, *rps15*, *ndhF*, *rpoC1*, *rps8*, *rpl20*, *rps18,* and *matK*) (Fig. 5A). These values of the non-coding regions ranged from 0 to 0.04122 (*trnH-GUG-psbA*) with an average value of 0.01057 (Fig. 5B). A total of five mutational hotspots that showed high values of PI (≥ 0.02) were identified, including *trnH-GUG-psbA*, *rps15-ycf1*, *trnG-GCC-trnR-UCU*, *trnC-GCA-petN*, and *ndhF-rpl32*. The analysis revealed that the protein-coding regions exhibit lower divergence levels than non-coding regions. These hotspot regions could be utilized as potential molecular markers for phylogenetic studies and the identification of *Rhodiola* species.

### Phylogenetic analysis within *Rhodiola*

**Fig. 6:**
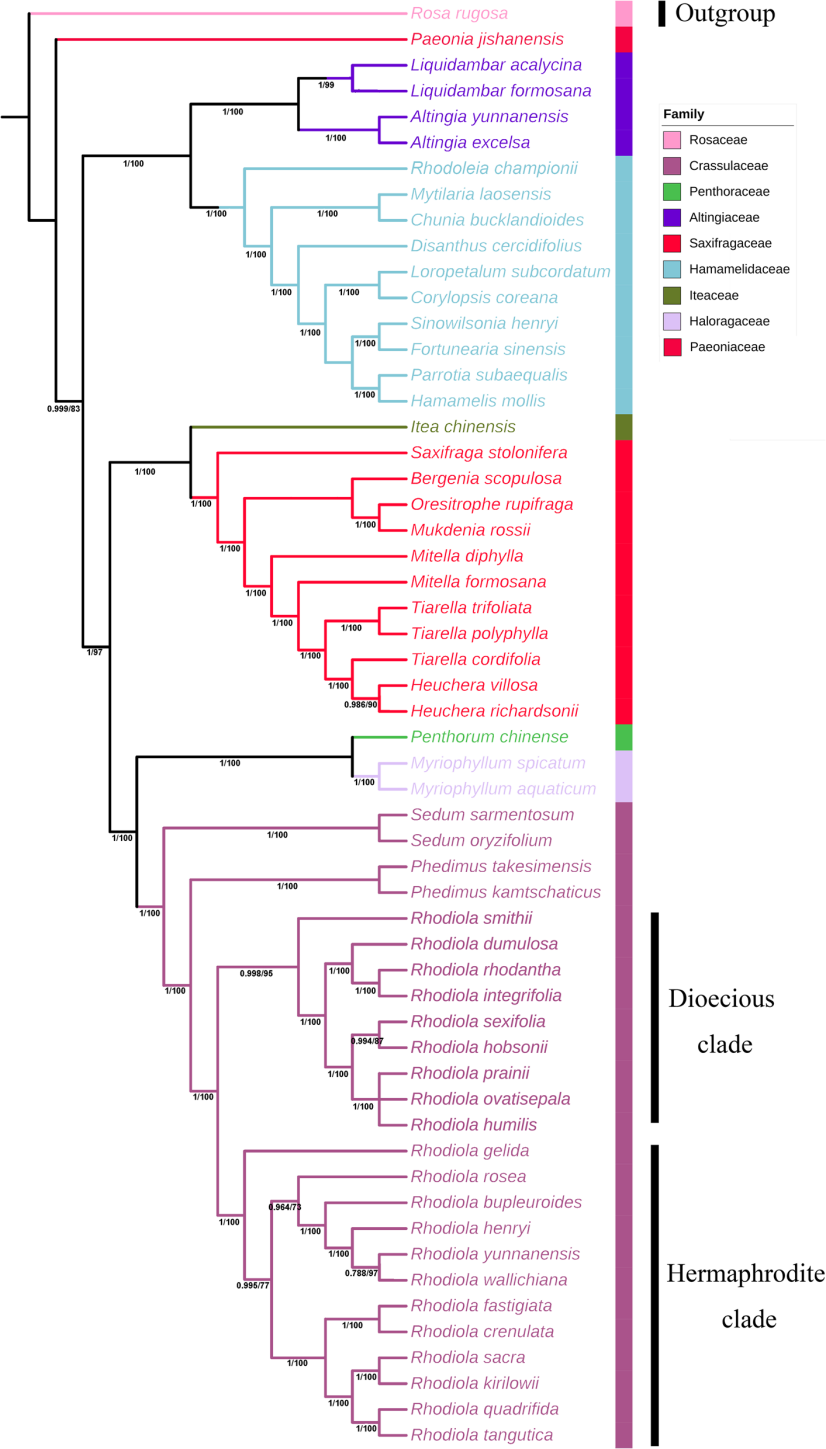
Phylogenetic tree based on 38 protein-coding genes shared by the cp genomes of 55 Saxifragales species. The tree was generated using a ML method with 1000 bootstrap replicates. Numbers on the nodes indicate bootstrap values. Different colors represent species belonging to different families

Complete cp genomes comprise abundant phylogenetic information, which could be applied to evolution and phylogenetic studies of angiosperms because of several advantages, such as high accuracy and resolution [22]. In order to clarify the phylogenetic position of six newly assembled *Rhodiola* within the Saxifragales, phylogenetic tree was constructed. The phylogenetic tree showed that all *Rhodiola* species formed a monophyletic clade and then were classified into two separate branches (Fig. 6), which is inconsistent with the record in Flora of China [1]. The dioecious clade composed of nine dioecious *Rhodiola* species, and the hermaphrodite clade included all the hermaphrodite species.

## Materials and methods

All methods were performed in accordance with the relevant guidelines and regulations.

### Plant materials and DNA extraction

Wild plant materials of *Rhodiola* were collected in Nyingchi (Tibet, China). The specimens of *Rhodiola* have been kept at the Tibet Agriculture & Animal Husbandry University and Kunming Institute of Botany. Total genomic DNA was extracted from silica-gel-dried leaves using the modified CTAB (cetyltrimethylammonium bromide) method [23]. The quantity and quality of extracted genomic DNA were determined by gel electrophoresis and NanoDrop 2000 Spectrophotometer (Thermo Scientific, Carlsbad, CA, USA).

### Chloroplast genome sequencing, assembly and annotation

Genomic DNA was randomly fragmented by sonication (Covaris, M220). Paired-end sequencing libraries were constructed according to the Illumina standard protocol (Illumina, San Diego, CA, USA). Sample sequencing was carried out on an Illumina Hiseq X-Ten platform. Total genomic DNAs were also sent to BGI (Shenzhen, China) for library (400 bp) preparation for genome skimming sequencing. Paired-end (150 bp) sequencing was conducted on the Illumina HiSeq X-10 platform, generating ∼2 Gb data per sample. Raw reads were filtered by quality control software NGS QC Toolkit v2.3.333 to obtain high quality Illumina data [24].

Next, filtered reads were de novo assembled using NOVOPlasty [25] with parameters of K-mer (33). These assembled chloroplast genomes were annotated in GeSeq [26], coupled with manually edited start and stop codons in Geneious 11.1.4 [27] (Biomatters Ltd., Auckland, New Zealand) with a reference *R. rosea* chloroplast genome (Genbank accession number MH410216). In addition, all tRNA genes were further verified using tRNAscan-SE v1.21 [28]. The border region between the inverted repeat (IR) and the large single copy (LSC), also between inverted repeats and small single copy (SSC) junction were determined through local BLAST software. Finally, the circular gene maps of *Rhodiola* plastomes were drawn utilizing the Organellar Genome DRAW tool (OGDRAW) [29].

### Comparative genomic analysis and molecular marker identification

To detect variations within *Rhodiola* cp genomes, we compared the cp genomes of *R. crenulata*, *R. rosea,* and the six newly assembled *Rhodiola* cp genomes by mVISTA [30]. The nucleotide diversity of the *Rhodiola* cp genomes was detected by DNA Sequence Polymorphism (DnaSP) software [31].

### Characterization of repeat sequence and SSRs

The long repetitive sequences were detected using REPuter with a 30 bp minimum repeat size and a Hamming distance of 3 [32]. Simple sequence repeats (SSRs) in the cp genomes were identified via the MISA perl script [33] with the minimum number of repeats set to 8, 5, 3, 3, 3 and 3 for mono-, di-, tri-, tetra-, penta-, and hexa-nucleotides, respectively.

### Phylogenetic analysis

The phylogenetic analysis was performed on six newly assembled *Rhodiola* cp genomes, another 55 Saxifragales species, and one outgroup *Rosa rugosa*, all of which were down loaded from the NCBI (Table S4) except those of six newly assembled *Rhodiola* cp genomes. Molecular phylogenetic trees, using aligned sequences of 38 protein-coding genes (Table S5) with MAFFT 7.0 [34] and adjusted manually where necessary, were constructed using IQ-TREE (Nguyen et al.,2015) and MrBayes 3.2.6 software [35] under the GTRGAMMA model.

## Discussion

Six *Rhodiola* cp genomes were sequenced and assembled in our study, and this information was used to identify candidate DNA markers and infer *Rhodiola* phylogeny. The size of the cp genome, the length of the SSC, LSC, and IR regions, the content of the GC, and gene content demonstrated a high degree of similarity among the genomes, implying that *Rhodiola* species shared low diversity [4]. The IR region, however, has a larger GC content than the LSC and SSC regions. In most angiosperm chloroplasts, there are 74 protein-coding genes, with an additional five in a few species [36]. Six newly assembled *Rhodiola* cp genomes contain 85 protein-coding genes, 37 tRNA genes, and 8 rRNA genes, which is consistent with previous studies [4].

Repetitive sequences play crucial roles in chloroplast genome arrangement and sequence divergence [37]. Reverse repeats and complementary repeats only exist in the cp genome of *R. gelida,* reflecting the fact that *Rhodiola* chloroplast genomes exhibited a significant difference in type, length and number of repeats. Chloroplast SSR markers are efficient genetic resources to investigate population genetics and biogeography of closely related taxa due to their relatively richness, high reproducibility and polymorphism [38, 39]. Wang et al. used 11 ISSR primers to reveal the interspecific or intraspecific genetic differences and diversity of four *Rhodiola* species [9].In our study, A and T nucleotides were the most common, while tandem G or C repeats were quite rare (Fig. 3A), which was in concordance with the other research results [40, 41].These SSR markers could be used to examine the genetic structure, differentiation, diversity, and maternity in the 6 *Rhodiola* species and their relative species in future studies.

The whole cp genome contains abundant mutation sites, which can be used directly as a super barcode for species identification. As with hypervariable regions of the genome, they can also be screened out as potential molecular markers [42, 43]. At present, many species have been successfully identified based on the chloroplast genome, especially the species with frequent hybridization and apomixis [43–45]. *Ycf1*, *rps15*, *ndhF*, *rpoC1*, *rps8*, *rpl20*, *rps18* and *matK* genes in CDS showed significant variation and high sequence variations were found in intergenic regions as follows: *trnH-GUG-psbA*, *rps15-ycf1*, *trnG-GCC-trnR-UCU*, *trnC-GCA-petN*, and *ndhF-rpl32* (Fig. 6)*.* These regions can also be used as candidate markers for elucidating the phylogenetic relationship among *Rhodiola* species. In many species, *TrnH-GUG-psbA* and *matK* are the most mutated hotspots for species identification, such as *Kengyilia* [46], Apocynaceae [47] and Orchidinae [48]. *Ycf1* marker has good species identification resolution in *Pinus* at the within-genus level relationships [49]. *Ycf1* has a better effect on the identification of *Rhodiola* due to its longer sequence (~ 5800 bp). We recommend that the *ycf1* gene be used to reconstruct phylogenetic relationships of *Rhodiola* where there is a lack of genomic information.

Our phylogenetic analysis strongly supported the monophyly of *Rhodiola* species, which is consistent with previous studies [3, 4]. All *Rhodiola* species are classified into two separate branches (dioecious and hermaphrodite), which supports the view of Zhao et al. [4]. What interests us is that *R. wallichiana* was gathered in the hermaphrodite clade, and we think that we may have selected a rare unisexualis among *R. wallichiana*. So, we speculate that unisexual *R. wallichiana* and bisexual *R. wallichiana* may have huge genetic differences. In addition, the dioecious clade also contains *R. integrifolia*, which has been temporarily classified as a hybrid between *R. rosea* and *R. rhodantha* [50]*.* Our phylogenetic analyses also revealed that there are close relationships between Crassulaceae and Saxifragaceae, supporting the view that there is a common origin between them. In some traditional angiosperm classification systems, Saxifragaceae is the largest group of garbage bins in angiosperms, and many branches that are unrelated in evolution are forcibly pieced together into a highly multi-line group. Our phylogeny suggested that the Penthoraceae and Haloragidaceae were clustered into one clade, indicating their close relationship. The APGII research considers that Penthoraceae can be selectively combined with Haloragidaceae [51], our research seems to support this view. However, since there is only one chloroplast genome data in two families and lack of data on the cp genome of more species, we believe that when more species in the two families have been sequenced to accurately determine their evolutionary relationship.

## Conclusions

In this study, we determined and characterized six cp genome sequences of *Rhodiola*, which are commonly used as Tibetan medicinal materials. The size of the genome, the structure and organization of genes were shown to be conservative, which is similar to those reported cp genomes of *Rhodiola* species. To develop molecular markers for future phylogeographic and population genetic studies, thirteen mutational hotspots were identified*.* The results of phylogenetic analysis showed that *Rhodiola* species were clustered into two clades: dioecious and hermaphrodite, with strong support values. The complete cp genome sequences that were newly assembled facilitate medicinal resource conservation, phylogenetic reconstruction, and biogeographical research of *Rhodiola*.

## Supplementary Information

**Additional file 1: Table S1.** Summary of six *Rhodiola* chloroplast genome characteristics.

**Additional file 2: Table S2.** List of genes in six* Rhodiola* chloroplast genomes.

**Additional file 3: Table S3.** List of species used to evaluate the sequence divergence.

**Additional file 4: Table S4.** List of species used for phylogenetic tree construction.

**Additional file 5: Table S5.** List of 38 protein-coding genes used for phylogenetic tree construction.

## Data Availability

The datasets supporting the results of this publication are included within the article and Additional files 1, 2, 3, 4, 5. The chloroplast genome data of 6 cp genome sequences of *Rhodiola* used for analysis could be obtained from NCBI, and their accession numbers are as follow: *R. wallichiana*, OL742458; *R. henryi*, OL742459; *R. gelida*, OL742460; *R. bupleuroides*, OL742461; *R. tangutica*, OL742462; *R. quadrifida*, OL742463. Data for our newly assembled six *Rhodiola* chloroplast genomes are also available in Supplementary S6. Voucher specimens of 6 *Rhodiola* were deposited in the Tibet Agriculture & Animal Husbandry University and Kunming Institute of Botany and identified by Professor Xiaozhong Lan, and their accession numbers are as follow: *R. wallichiana*, WH-2013–029; *R. henryi*, GanQL691; *R. gelida*, ChenSL1377; *R. bupleuroides*, LiuJQ-08XZ-062; *R. tangutica*, ChenSL0277; *R. quadrifida*, ChenSL0149.
