# Supplementary material for: Comparative analyses of chloroplast genomes from Six Rhodiola species: variable DNA markers identification and phylogenetic relationships within the genus
Source: BMC Genomics. 2022 Aug 11;23:577. doi: 10.1186/s12864-022-08834-9 (PMC9373441; doi:10.1186/s12864-022-08834-9)
Supplement: Supplementary file 2 — Additional file 2: Table S2. List of genes in six Rhodiola chloroplast genomes. [file 12864_2022_8834_MOESM2_ESM.docx]

**Table S2.** List of genes in six *Rhodiola* chloroplast genomes.

| **Groups of Genes** | **Names of Genes** |
| --- | --- |
| rRNA genes | *rrn16*(🞨2)*, rrn23*(🞨2)*, rrn4.5*(🞨2)*, rrn5*(🞨2) |
| tRNA genes | *trnA-UGC* (*🞨*2), trnC-GCA, trnD-GUC, trnE-UUC, trnF-GAA, trnfM-CAU, trnG-GCC*, trnG-UCC, trnH-GUG, trnI-CAU(*🞨*2), trnI-GAU* (*🞨*2), trnK-UUU*, trnL-CAA(*🞨*2), trnL-UAA*, trnL-UAG, trnM-CAU, trnN-GUU(*🞨*2), trnP-UGG, trnQ-UUG, trnR-ACG(*🞨*2), trnR-UCU, trnS-GCU, trnS-GGA, trnS-UGA, trnT-GGU, trnT-UGU, trnV-GAC(*🞨*2), trnV-UAC*, trnW-CCA, trnY-GUA* |
| Small subunit of ribosome | *rps11, rps12***(🞨2)*, rps14, rps15, rps16*, rps18, rps19*(🞨2)*, rps2, rps3, rps4, rps7*(🞨2)*, rps8* |
| Large subunit of ribosome | *rpl33, rpl36, rpl14, rpl16*, rpl20,rpl22, rpl2**(🞨2)*, rpl23*(🞨2)*, rpl32* |
| DNA-dependent RNA polymerase | *rpoC2, rpoC1*, rpoB, rpoA* |
| Subunit of Photosystem 1 | *psaB, psaA, psaC, psaI, psaJ* |
| Subunit of Photosystem 2 | *psbA, psbB, psbC, psbD, psbE, psbF, psbH, lhbA, psbI, psbJ, psbK, psbL, psbM, psbN, psbT,* |
| Subunits of cytochrome b/f complex | *petA, petB*, petD*, petG, petL, petN* |
| Subunits of ATP synthase | *atpA, atpB, atpE, atpF*, atpH, atpI* |
| Protease | *clpP*** |
| Large subunit of rubisco | *rbcL* |
| Subunit of NADH-dehydrogenase | *ndhA*, ndhB**(🞨2)*, ndhC, ndhD, ndhE, ndhF, ndhG, ndhH, ndhI, ndhJ, ndhK* |
| Maturase | *matK* |
| Envelope membrane protein | *cemA* |
| Subunit of Acetyl-CoA-carboxylase | *accD* |
| Synthesis gene | *ccsA* |
| Open reading frames (ORF, ycf) | *ycf1*(🞨2)*, ycf2*(🞨2)*, ycf3**, ycf4, ycf15*(🞨2) |

* indicates gene with one intron and ** indicates gene with two introns. (×2) indicates that the number of the repeat unit is 2. The rps12 gene is a trans-spliced gene.
