# Supplementary material for: Comparative analyses of chloroplast genomes from Six Rhodiola species: variable DNA markers identification and phylogenetic relationships within the genus
Source: BMC Genomics. 2022 Aug 11;23:577. doi: 10.1186/s12864-022-08834-9 (PMC9373441; doi:10.1186/s12864-022-08834-9)
Supplement: Supplementary file 3 — Additional file 3: Table S3. List of species used to evaluate the sequence divergence. [file 12864_2022_8834_MOESM3_ESM.docx]

**Table S3.** List of species used to evaluate the sequence divergence.

|  | Species | GenBank Number |
| --- | --- | --- |
| 1 | *Rhodiola bupleuroides* | OL742461 |
| 2 | *Rhodiola crenulata* | MN794322.1 |
| 3 | *Rhodiola dumulosa* | MN794323.1 |
| 4 | *Rhodiola fastigiata* | MN794324.1 |
| 5 | *Rhodiola gelida* | OL742460 |
| 6 | *Rhodiola henryi* | OL742459 |
| 7 | *Rhodiola hobsonii* | MN794325.1 |
| 8 | *Rhodiola humilis* | MN794326.1 |
| 9 | *Rhodiola integrifolia* | MN794327.1 |
| 10 | *Rhodiola kirilowii* | MN109979.1 |
| 11 | *Rhodiola ovatisepala* | MN794328.1 |
| 12 | *Rhodiola prainii* | MN794329.1 |
| 13 | *Rhodiola quadrifida* | OL742463 |
| 14 | *Rhodiola rhodantha* | MN794330.1 |
| 15 | *Rhodiola rosea* | NC_041671.1 |
| 16 | *Rhodiola sacra* | MN109978.1 |
| 17 | *Rhodiola sexifolia* | MN109980.1 |
| 18 | *Rhodiola smithii* | MN794331.1 |
| 19 | *Rhodiola tangutica* | OL742462 |
| 20 | *Rhodiola wallichiana* | OL742458 |
| 21 | *Rhodiola yunnanensis* | MN794332.1 |
| 22 | *Rhodoleia championii* | NC_045276.1 |
