# Supplementary material for: Comparative analyses of chloroplast genomes from Six Rhodiola species: variable DNA markers identification and phylogenetic relationships within the genus
Source: BMC Genomics. 2022 Aug 11;23:577. doi: 10.1186/s12864-022-08834-9 (PMC9373441; doi:10.1186/s12864-022-08834-9)
Supplement: Supplementary file 4 — Additional file 4: Table S4. List of species used for phylogenetic tree construction. [file 12864_2022_8834_MOESM4_ESM.docx]

**Table S4.** List of species used for phylogenetic tree construction.

|  | Species | GenBank Number |
| --- | --- | --- |
| 1 | *Altingia excelsa* | NC_048995.1 |
| 2 | *Altingia yunnanensis* | NC_048981.1 |
| 3 | *Bergenia scopulosa* | NC_036061.1 |
| 4 | *Cercidiphyllum japonicum* | NC_037940.1 |
| 5 | *Cercidiphyllum magnificum* | NC_046692.1 |
| 6 | *Chunia bucklandioides* | NC_041163.1 |
| 7 | *Corylopsis coreana* | NC_040141.1 |
| 8 | *Disanthus cercidifolius* | NC_050371.1 |
| 9 | *Fortunearia sinensis* | NC_041487.1 |
| 10 | *Hamamelis mollis* | NC_037881.1 |
| 11 | *Itea chinensis* | NC_037884.1 |
| 12 | *Liquidambar acalycina* | NC_046936.1 |
| 13 | *Liquidambar formosana* | NC_023092.1 |
| 14 | *Loropetalum subcordatum* | NC_037694.1 |
| 15 | *Mitella diphylla* | NC_042925.1 |
| 16 | *Mitella formosana* | NC_042926.1 |
| 17 | *Mukdenia rossii* | NC_037495.1 |
| 18 | *Mytilaria laosensis* | NC_048997.1 |
| 19 | *Myriophyllum aquaticum* | NC_048889.1 |
| 20 | *Myriophyllum spicatum* | NC_037885.1 |
| 21 | *Oresitrophe rupifraga* | NC_037514.1 |
| 22 | *Paeonia jishanensis* | NC_050330.1 |
| 23 | *Parrotia subaequalis* | NC_037243.1 |
| 24 | *Penthorum chinense* | NC_023086.1 |
| 25 | *Phedimus kamtschaticus* | NC_037946.1 |
| 26 | *Phedimus takesimensis* | NC_026065.1 |
| 27 | *Rhodiola bupleuroides* | OL742461 |
| 28 | *Rhodiola crenulata* | MN794322.1 |
| 29 | *Rhodiola dumulosa* | MN794323.1 |
| 30 | *Rhodiola fastigiata* | MN794324.1 |
| 31 | *Rhodiola gelida* | OL742460 |
| 32 | *Rhodiola henryi* | OL742459 |
| 33 | *Rhodiola hobsonii* | MN794325.1 |
| 34 | *Rhodiola humilis* | MN794326.1 |
| 35 | *Rhodiola integrifolia* | MN794327.1 |
| 36 | *Rhodiola kirilowii* | MN109979.1 |
| 37 | *Rhodiola ovatisepala* | MN794328.1 |
| 38 | *Rhodiola prainii* | MN794329.1 |
| 39 | *Rhodiola quadrifida* | OL742463 |
| 40 | *Rhodiola rhodantha* | MN794330.1 |
| 41 | *Rhodiola rosea* | NC_041671.1 |
| 42 | *Rhodiola sacra* | MN109978.1 |
| 43 | *Rhodiola sexifolia* | MN109980.1 |
| 44 | *Rhodiola smithii* | MN794331.1 |
| 45 | *Rhodiola tangutica* | OL742462 |
| 46 | *Rhodiola wallichiana* | OL742458 |
| 47 | *Rhodiola yunnanensis* | MN794332.1 |
| 48 | *Rhodoleia championii* | NC_045276.1 |
| 49 | *Rosa rugosa* | MK986659.1 |
| 50 | *Saxifraga stolonifera* | NC_037882.1 |
| 51 | *Sedum oryzifolium* | NC_027837.1 |
| 52 | *Sedum sarmentosum* | NC_023085.1 |
| 53 | *Sinowilsonia henryi* | NC_036069.1 |
| 54 | *Tiarella cordifolia* | NC_042927.1 |
| 55 | *Tiarella polyphylla* | NC_042928.1 |
| 56 | *Tiarella trifoliata* | NC_042929.1 |
