# Supplementary material for: Comparative analyses of chloroplast genomes from Six Rhodiola species: variable DNA markers identification and phylogenetic relationships within the genus
Source: BMC Genomics. 2022 Aug 11;23:577. doi: 10.1186/s12864-022-08834-9 (PMC9373441; doi:10.1186/s12864-022-08834-9)
Supplement: Supplementary file 5 — Additional file 5: Table S5. List of 38 protein-coding genes used for phylogenetic tree construction. [file 12864_2022_8834_MOESM5_ESM.docx]

**Table S5.** List of 38 protein-coding genes used for phylogenetic tree construction.

|  | Genes |  | Genes |
| --- | --- | --- | --- |
| 1 | *accD* | 20 | *petA* |
| 2 | *atpA* | 21 | *petB* |
| 3 | *atpB* | 22 | *petG* |
| 4 | *atpE* | 23 | *petL* |
| 5 | *atpF* | 24 | *petN* |
| 6 | *atpH* | 25 | *psaA* |
| 7 | *atpI* | 26 | *psaB* |
| 8 | *ccsA* | 27 | *psaC* |
| 9 | *clpP* | 28 | *psaJ* |
| 10 | *matK* | 29 | *psbA* |
| 11 | *ndhA* | 30 | *psbC* |
| 12 | *ndhB* | 31 | *psbD* |
| 13 | *ndhC* | 32 | *psbF* |
| 14 | *ndhE* | 33 | *psbH* |
| 15 | *ndhF* | 34 | *psbJ* |
| 16 | *ndhG* | 35 | *psbK* |
| 17 | *ndhH* | 36 | *psbN* |
| 18 | *ndhI* | 37 | *psbT* |
| 19 | *ndhJ* | 38 | *rbcL* |
